# HMB in DRAM-less NVMe SSDs: Their usage and effects on performance

**DOI:** 10.1371/journal.pone.0229645

**Published:** 2020-03-02

**Authors:** Kyusik Kim, Taeseok Kim

**Affiliations:** 1 Department of Computer Engineering, Kwangwoon University, Nowon-gu, Seoul, Republic of Korea; 2 School of Computer and Information Engineering, Kwangwoon University, Nowon-gu, Seoul, Republic of Korea; King Abdulaziz University, SAUDI ARABIA

## Abstract

Solid-state drives (SSDs) that do not have internal dynamic random-access memory (DRAM) are being widely spread for client SSD and embedded SSD markets in recent years because they are cheap and consume less power. Obviously, their performance is lower than conventional SSDs because they cannot exploit advantages of DRAM in the controller. However, this problem can be alleviated by using host memory buffer (HMB) feature of Non-Volatile Memory Express (NVMe), which allows SSDs to utilize the DRAM of host. In this paper, we show that commercial DRAM-less SSDs clearly exhibit worse I/O performance than SSDs with internal DRAM, but this can be improved by using the HMB feature. We also present methods that reveal how the host memory buffer is used in commercial DRAM-less SSDs to improve I/O performance. Through extensive experiments, we conclude that DRAM-less SSDs evaluated in this study mainly exploit the host memory buffer as an address mapping table cache rather than a read cache or write buffer to improve I/O performance.

## Introduction

For the last decade, almost solid-state drives (SSDs) have included dynamic random-access memory (DRAM) in their controller to improve input/output (I/O) performance and endurance. DRAM in the controller is usually used for temporarily keeping data that have been read from flash memory, data to be written to flash memory, or an address mapping table, and it has been regarded as a necessary component of SSDs [1–5, 6]. Contrary to this convention, some companies have recently brought to market DRAM-less SSDs that do not contain DRAM in the controller. If DRAM is eliminated from the controller, the power consumption, manufacturing cost, and size of the form factor can decrease [7–9]. For this reason, the market of DRAM-less SSDs has been rapidly increasing in customer, embedded, and enterprise SSDs and they have been considered as storage for distributed edge computing due to the reduced size [10–12]. Unfortunately, DRAM-less SSDs also have clear disadvantages. Because DRAM in the controller cannot be used for caching data read/written or address mapping table, I/O performance is unavoidably degraded.

**Fig 1:**
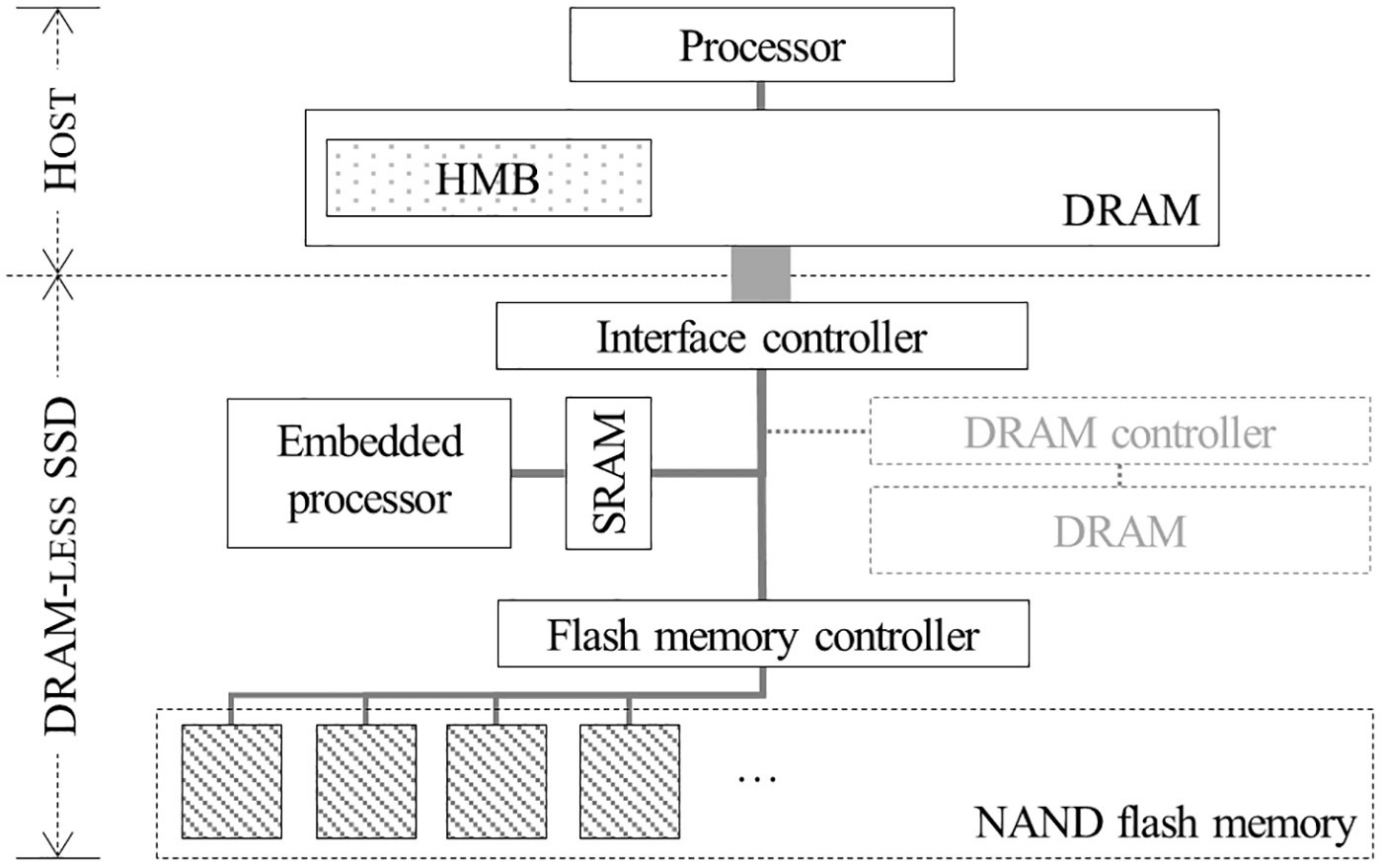
Architecture of DRAM-less SSD supporting HMB.

For DRAM-less SSDs with a Non-Volatile Memory Express (NVMe) interface, this problem can be alleviated by using the host memory buffer (HMB) feature of NVMe [9, 13]. The HMB is a feature introduced in the NVMe 1.2 protocol, which allows an SSD to utilize the DRAM of the host for its own purposes [14]. As the controller can access the host DRAM through very fast Peripheral Component Interconnect Express (PCIe)/NVMe interface, it can exploit a portion of host DRAM as a cache for data or address mapping table as if it accesses DRAM in the controller (Fig 1). In the last few years, DRAM-less SSD controllers and DRAM-less SSDs that support the HMB feature have been developed and shipped, and operating systems exploiting these devices have also been studied [15–18].

Although the use of HMB is a main approach to reduce the I/O performance degradation of DRAM-less SSDs, we do not know exactly how to utilize the host DRAM with the HMB feature in DRAM-less SSDs because this is a trade secret. In this paper, we first experimentally confirm that current commercial DRAM-less SSDs have poorer I/O performance than SSDs with DRAM in their controllers but they improve the I/O performance to some extent when using the HMB. We also present methods for experimentally understanding how DRAM is used in commercial DRAM-less SSDs supporting HMB. Assuming that the HMB can be used as 1) a read cache, 2) a write buffer, or 3) an address mapping table cache, we present methods that can test the existence for all these cases. Through extensive experiments, we conclude that DRAM-less SSDs evaluated in our study utilize most parts of the host DRAM as a cache for storing an address mapping table.

The remainder of this paper is organized as follows. First, we describe the background and related work in Section II. Then, we analyze how much the I/O performance degrades when DRAM is eliminated from the SSD controller as well as how commercial DRAM-less SSDs utilize the HMB in Sections III and IV, respectively. Finally, we make our concluding remarks in Section V.

## Background and related work

### HMB feature of NVMe interface

NVMe is a high-performance scalable host controller interface designed for non-volatile memories such as PCIe based SSDs [19]. To provide high speed I/O, it supports up to 65,535 submission and completion queues that can queue up to 64K commands [4, 14, 20, 21]. Due to the scalable architecture, the internal parallelism of SSDs can be fully exploited. I/O requests issued by the host are delivered to submission queues, and then the SSD device dispatches them from the submission queues in a round-robin fashion. After processing the I/O requests in the SSD device, the messages notifying the I/O completion are inserted to the completion queues.

The NVMe interface also provides additional features to improve the performance of SSDs effectively. One of them is HMB [14], which was added in the NVMe 1.2 specification and allocates a portion of the host DRAM for the SSD. The usage of the memory space is determined by the SSD manufacturers. If both the SSD and host’s operating system support the HMB feature, it can be activated by the *set features* command, which is an NVMe administration command sent from the host’s operating system to the SSD. In the latest Linux kernel, the NVMe device driver attempts to activate the HMB during device initialization if the NVMe SSD device supports it [15].

In DRAM-less SSDs, the HMB feature provides several opportunities for alleviating the I/O performance degradation. The NVMe interface provides very fast data transmission speeds between the host and SSD controller, so the SSD controller can access the host DRAM without performance loss, that is, as if the controller were using its own DRAM. In addition, because the host DRAM can be accessed from the host’s operating system as well as the SSD controller, more benefits could be obtained if it were effectively used. Nonetheless, there seem to be few studies on improving I/O performance using HMB in DRAM-less SSDs. In [13], authors used a host DRAM of 128 MB for caching an address mapping table in DRAM-less SSDs. They demonstrated that enabling the HMB boosts the input/output operations per second (IOPS) performance significantly compared to other DRAM-less solutions.

In [22], Hong et al. used a host DRAM as a data cache instead of an address mapping table cache by modifying the NVMe command process and adding a direct memory access (DMA) path between system memory and the host DRAM. The proposed scheme improved the I/O performance by 23% for sequential writes compared to an architecture with internal DRAM in the SSD. In [23], although the HMB feature of NVMe is not directly used, Jeong et al. proposed a scheme called the host performance booster (HPB) in which a portion of the host DRAM is used as an address mapping table cache. They define transactional protocols between the host device driver and storage device to manage the address mapping table cache in the host. By implementing the HPB on smartphones with a UFS (Universal Flash Storage) device, they showed a performance improvement up to 67% for random read workloads.

### Internal SSD information for optimization

Because of the fixed device interfaces, host operating systems cannot know the internal information of SSDs such as buffer size and physical read/write sizes. The NVMe interface provides some commands that deliver the internal information of SSD to the host, such as *identify* and *get features* commands, but all SSDs do not implement such commands as well as information provided by the commands is still limited. An alternative is the Open-Channel SSD that migrates most of SSD’s internal operations to the host [24, 25]. Since the host directly performs the flash translation layer (FTL) operations with the internal information of the SSD, I/O requests can be more efficiently handled. For example, González et al. improved the I/O latency on a system supporting multi-tenancy by dividing the SSD into physical units and allowing the host to access those units in parallel [26]. However, since Open-Channel SSDs do not follow the traditional I/O stack, it requires supports of operating systems, and as a result, the utilization is greatly limited yet.

There are some studies that obtain the internal information of an SSD in an operating system by measuring the I/O performance with different workloads. Kim et al. extracted basic internal SSD information such as read cache size, write buffer size, cluster block size, and cluster page size by analyzing the times taken to process various workloads with regular patterns [27]. Using a similar approach, Ko et al. obtained the cluster page and cluster block sizes of an SSD and then used them to tune the operating system’s configuration parameters such as file system block size and I/O request size [28]. Using the SSD’s internal information, they also proposed an I/O scheduler that improves average I/O latency. There have also been studies that measure the performance of SSDs through exhaustive experiments. As a representative example, Jung et al. observed some interesting behaviors that contradict commonly held conceptions [29]. For example, they reported that the random read performance of an SSD is worse than both sequential and random write performance and that sequential reads become significantly worse over time. These studies were performed using older SATA SSDs. In this paper, we focus on the performance of NVMe SSDs, especially those that do not have any DRAM in the controller.

## I/O Performance of DRAM-less SSDs

To analyze how much the I/O performance of DRAM-less SSDs is degraded when compared to that of SSDs with internal DRAM, we evaluated six SSDs described in Table 1 using the fio benchmark tool [30]. SSD-A, SSD-B, and SSD-C are DRAM-less SSDs supporting the HMB feature and are all products that currently can be obtained commercially. SSD-D, SSD-E, and SSD-F are SSDs with internal DRAM and were chosen for the performance comparison. SSD-A, SSD-C, and SSD-D have similar hardware compositions except that SSD-A and SSD-C do not have any DRAM in the controller. SSD-B and SSD-E also have similar hardware compositions. Finally, SSD-F has the best hardware specification of all the SSDs compared in this study. In SSD-A, SSD-B, and SSD-C, the host DRAM size for HMB can be determined through NVMe device driver to within a limited range specified by each SSD. All experiments were performed in the PC environment listed in Table 2.

**Table 1:** Tested SSDs.

	SSD-A	SSD-B	SSD-C	SSD-D	SSD-E	SSD-F
Product	SP A80	HP EX900	Tammuz M730	Kingston A1000	WD Black 3D	Samsung 970 PRO
Interface	PCIe 3.0 x2	PCIe 3.0 x4	PCIe 3.0 x2	PCIe 3.0 x2	PCIe 3.0 x4	PCIe 3.0 x4
Controller DRAM	DRAM-less	DRAM-less	DRAM-less	O, size unknown	512MB	512MB
NAND Flash	3D TLC	3D TLC	3D TLC	3D TLC	3D TLC	3D MLC
Capacity	512GB	500GB	512GB	480GB	500GB	512GB
HMB	O (8MB to 480MB)	O (64MB only)	O (8MB to 480MB)	X	X	X

**Table 2:** Host PC environment.

Category	Description
Processor	Intel i7-8700 3.2GHz
Main memory	DDR4 16GB
OS	Ubuntu 16.04.4 (Kernel 4.13.10)
Benchmark tool	fio-2.2.10

**Fig 2:**
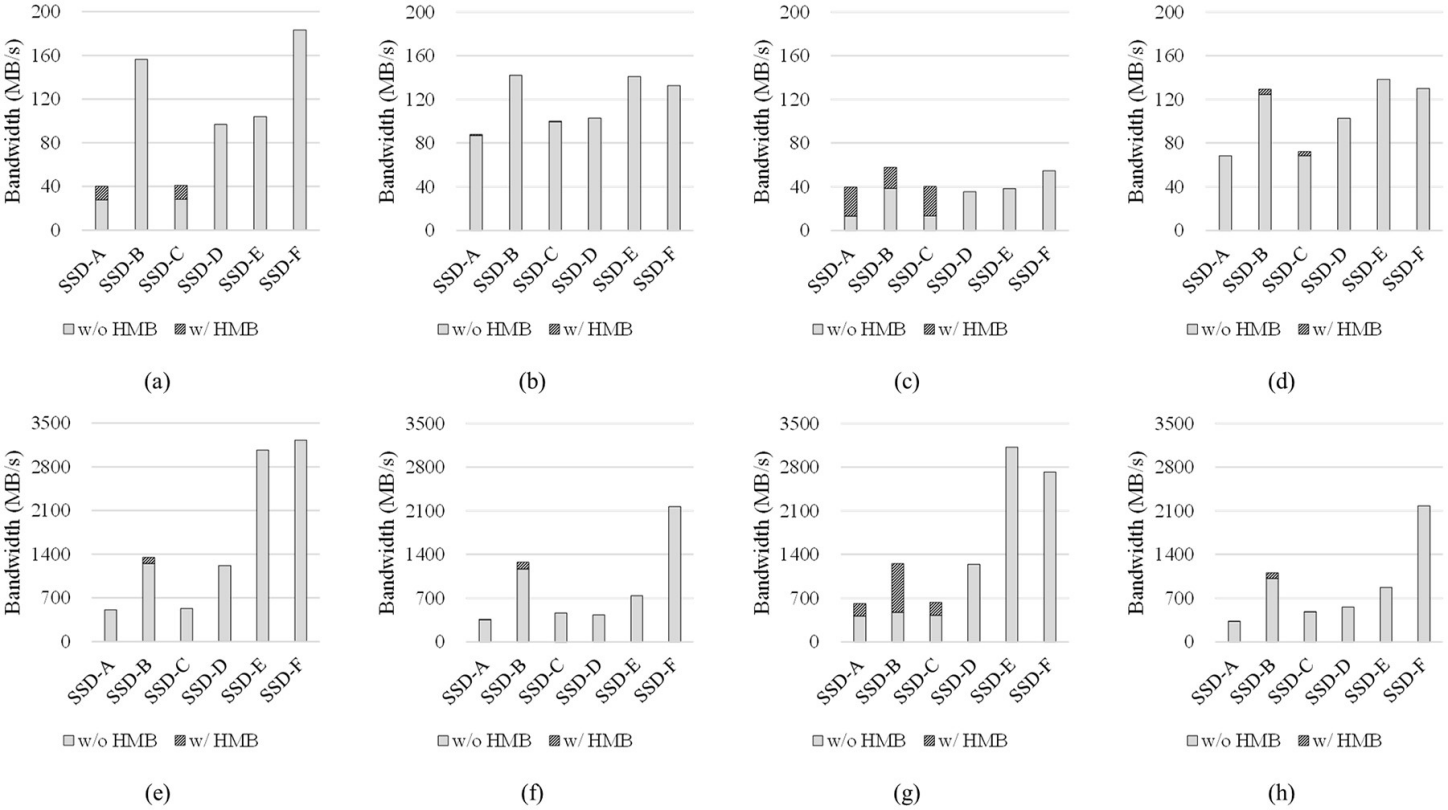
I/O performance comparison of DRAM-less SSDs (SSD-A, SSD-B, SSD-C) and SSDs with internal DRAM (SSD-D, SSD-E, SSD-F). (a) LIGHT, sequential read, (b) LIGHT, sequential write, (c) LIGHT, random read, (d) LIGHT, random write, (e) HEAVY, sequential read, (f) HEAVY, sequential write, (g) HEAVY, random read, and (h) HEAVY, random write.

We measured the I/O performances of these SSDs with two I/O workloads, LIGHT and HEAVY, which are created by configuring the fio benchmark to generate light and heavy workloads, respectively (Table 3). The experimental results are shown in Fig 2. In the results for the LIGHT workload (Fig 2(a)–2(d)), the lack of DRAM in the controller incurs a significant performance degradation in all experiments. Although SSD-A, SSD-C, and SSD-D have a similar hardware composition, when the HMB is disabled, SSD-A and SSD-C, which are DRAM-less SSDs, have much worse I/O performance than SSD-D. On the contrary, SSD-B achieves an I/O performance that is similar to that of SSD-E, which has almost the same hardware composition as SSD-B, when HMB is disabled.

**Table 3:** Two workloads by fio.

Parameter	LIGHT workload	HEAVY workload
#active CPU cores	1	12
#running threads	1	72
Total I/O size	16GB	100% of each SSD
Block size	4KB	4KB

In the results obtained for the HEAVY workload (Fig 2(e)–2(h)), DRAM-less SSDs have much worse I/O performance than SSDs with internal DRAM. Specifically, the gap in read performances between the two types of SSDs is substantial. When HEAVY workloads with random and sequential read patterns are used, SSD-A and SSD-C have a much lower bandwidth than SSD-D. In contrast to the experimental results obtained using the LIGHT workload, even SSD-B has much less bandwidth than SSD-E when HMB is disabled (Fig 2(e) and 2(g)). Finally, SSD-F exhibits better I/O performance than other SSDs overall in our experiments.

Next, we measured the I/O performance of DRAM-less SSDs after the HMB was activated. In the experiments using the LIGHT workload, the I/O performances of SSD-A and SSD-C are improved when the I/O patterns are sequential read and random read, as shown in Fig 2(a) and 2(c). Specifically, the performances of sequential read and random read become almost similar when HMB is used. The performance of SSD-B is improved only when the workload pattern is random read (Fig 2(c)). When the HEAVY workload is used, the performance of random read is improved again in all the SSDs we evaluated (Fig 2(g)). As in previous results, the performance of random read becomes similar to that of sequential read in SSD-A, SSD-B, and SSD-C when HMB is used.

Because the internals of SSDs such as architecture and FTL algorithm are maintained as trade secrets, we cannot answer exactly why the performances of the compared SSDs are different just with these results. However, it is clear that DRAM-less SSDs have worse I/O performances than SSDs with internal DRAM in many results and the I/O performance can be improved by using the HMB feature of NVMe. In the next section, we investigate how the HMB is utilized in DRAM-less SSDs through additional experiments.

## Use of HMB in DRAM-less SSDs

Almost all modern SSDs use DRAM in their controller as a read cache or write buffer to improve the I/O performance and lifecycle of NAND flash memory [1, 3–5]. DRAM is also used to temporarily store the mapping table for address translation [31]. Based on these facts, we hypothesize that commercial DRAM-less SSDs might use the HMB as a read cache, write buffer, or mapping table cache as if they were using their own DRAM in the controller. In this section, we present methods that can analyze how commercial DRAM-less SSDs make use of the HMB and demonstrate the results through extensive experiments.

### Use of HMB as a read cache

First, we present a method that checks whether the HMB is used as a read cache or not by referencing a previous work [27]. A read cache in an SSD is used to improve the read performance by storing data temporarily. If the data requested for a read are not in the read cache or the amount of data is larger than that of the read cache, the data should be read from NAND flash memory and thus the latency of the read request will increase. Hence, we perform the experiment described below to identify whether the HMB is used as a read cache.

**Fig 3:**
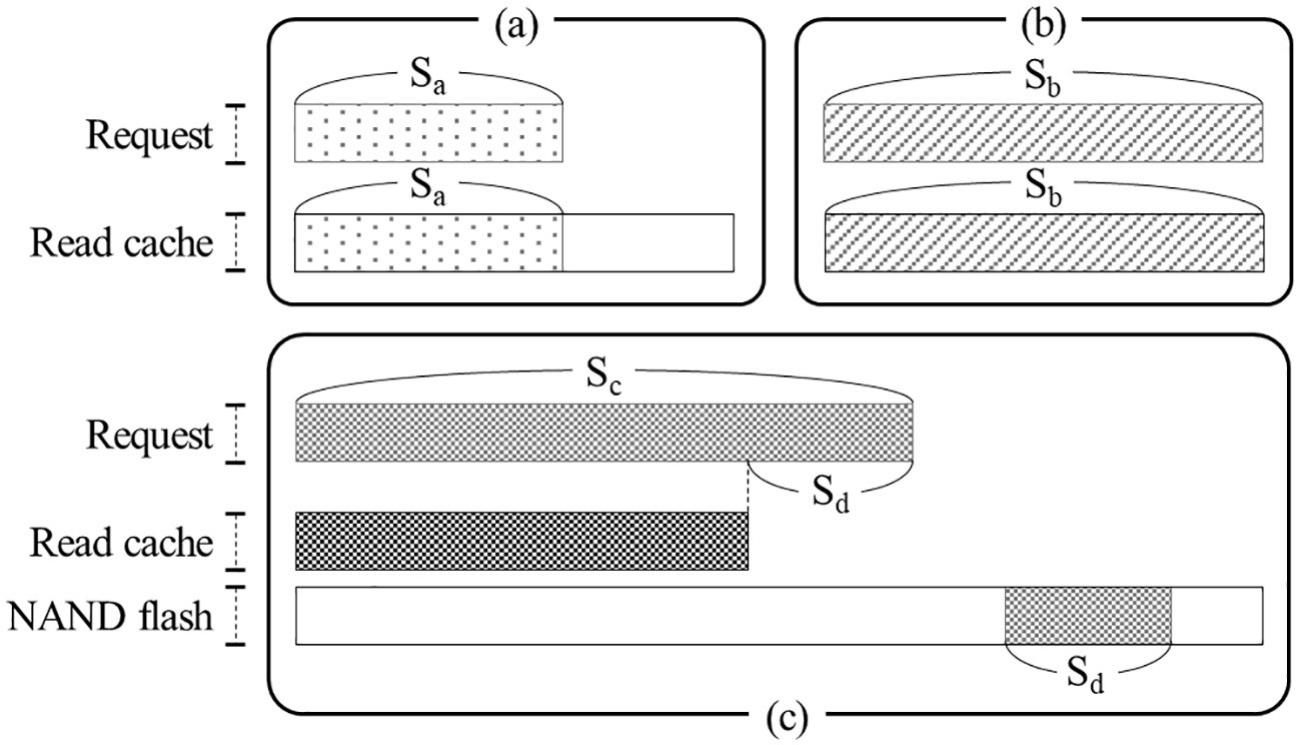
Some scenarios by read requests.

1. Open a raw DRAM-less SSD device with the O_DIRECT flag to bypass various caches and buffers in the operating system [32].
2. Read sufficient data from the SSD to completely fill a read cache if one exists (a maximum cache size of *S*_*CACHE*_). If the SSD has a read cache, the read cache will be filled with useless data.
3. Measure the time elapsed while reading data of size *s* from the SSD. If there is no read cache, the data should be read from NAND flash memory. Even if there is a read cache, because it is filled with useless data, *s*-sized data should be read from NAND flash memory and stored in the read cache.
4. Repeat step 3) and measure the elapsed time again. If there is a read cache, some or all of the requested data will be in the read cache. If the size of the requested data is smaller than or equal to that of the read cache, all the requested data will exist in the read cache (Fig 3(a) and 3(b)). In this case, the host will read all data only from the read cache in the HMB, so the read latency will be low. When the size of the requested data is larger than that of the read cache, part of the requested data, such as *S*_*d*_ in Fig 3(c), should be read from NAND flash memory, so the read latency will increase. Clearly, if a DRAM-less SSD does not use the HMB as a read cache, all data requested in this step will be read from NAND flash memory.
5. Repeat steps 2) to 4) while increasing the read request size *s* from *S*_*MIN*_ to *S*_*MAX*_ in increments of *S*_*INC*_. As mentioned above, the time taken to process the second read operation should sharply increase when *s* exceeds the read cache size.

**Fig 4:**
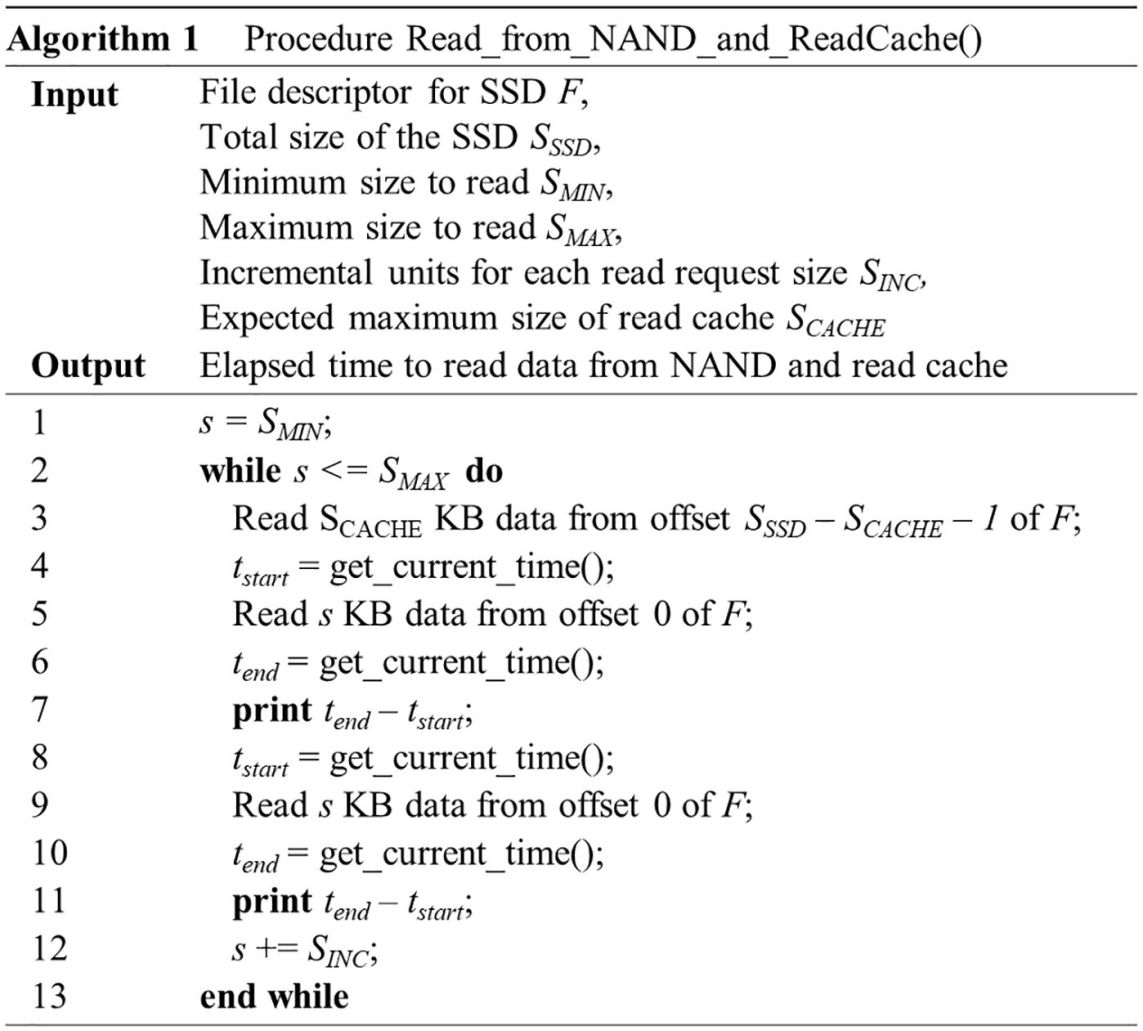
An algorithm for testing the existence of a read cache.

The pseudocode for this test is given in Fig 4. After setting *S*_*MIN*_ to 8 KB, *S*_*MAX*_ to 2,048 KB, and *S*_*INC*_ to 2 KB, we performed the above experiment on SSD-A, SSD-B, and SSD-C. The value of *S*_*CACHE*_ was set to 512 MB because these SSDs can be configured to use up to about 500 MB as the HMB space.

**Fig 5:**
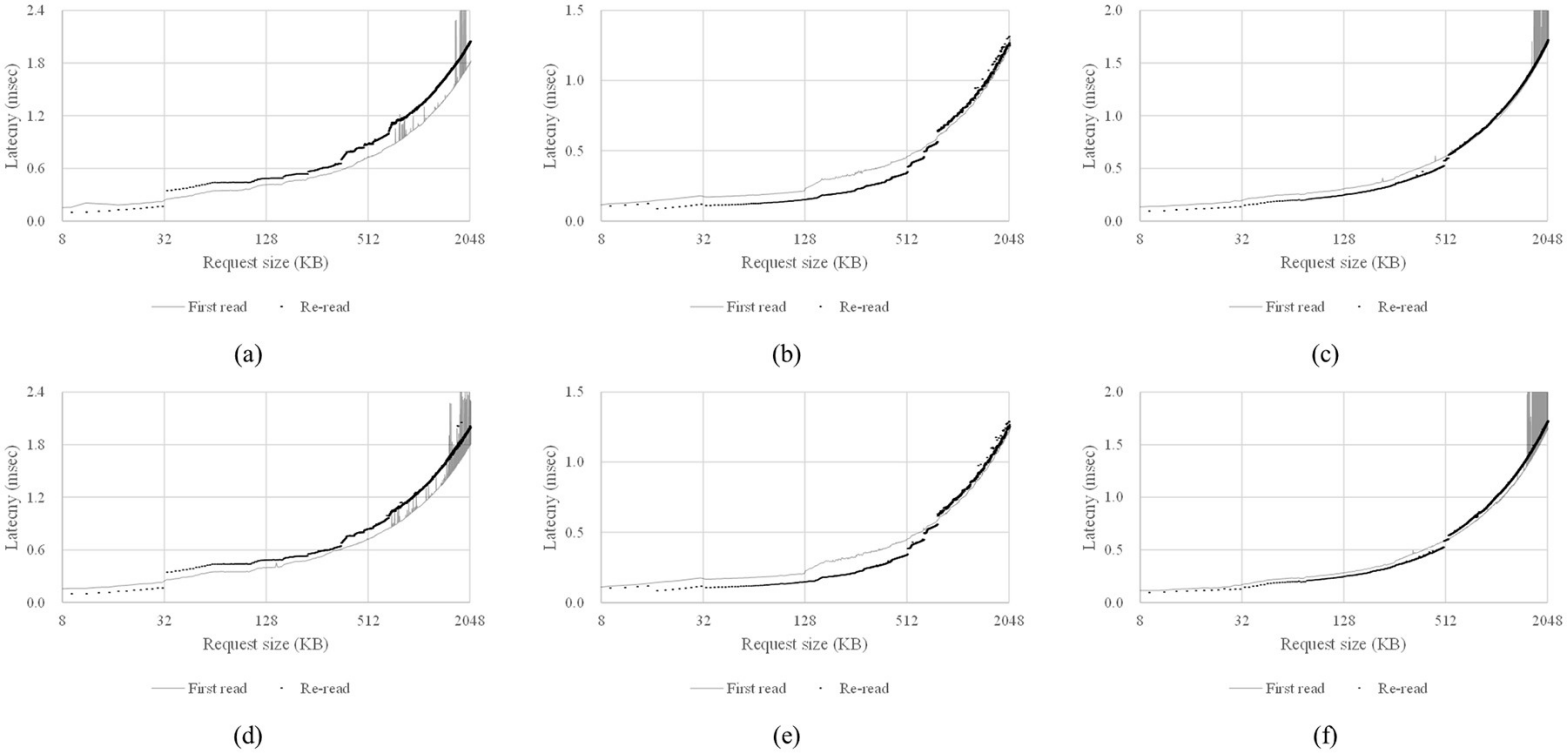
Test results for the existence of a read cache in the HMB of various SSDs. (a) SSD-A, with HMB, (b) SSD-B, with HMB, (c) SSD-C, with HMB, (d) SSD-A, without HMB, (e) SSD-B, without HMB, and (f) SSD-C, without HMB.

The results show that latencies of the re-read operations measured in step 4) increased suddenly when the read request sizes of SSD-A, SSD-B, and SSD-C were 32 KB, 768 KB, and 512 KB, respectively. When the request size is smaller than these values, re-read operations have lower latency than the first read operations because all the data requested can be accessed from the read cache instead of NAND flash memory. Note that this behavior is not different even when the HMB is not activated. As can be seen in Fig 5(d)–5(f), the curves in the graphs when HMB is not used are similar to previous results. It can be concluded that the DRAM-less SSDs used in our experiments employ some sort of read cache, but they use other media within the controller such as single level cell (SLC) NAND flash memory instead of the HMB as a read cache.

### Use of HMB as a write buffer

A write buffer in an SSD can improve the write performance and the lifecycle by storing the updated data into the controller’s DRAM temporarily. If the size of the updated data is larger than the write buffer size, part of the data should be flushed to NAND flash memory. Hence, we present the following method that determines whether the HMB is used as a write buffer or not by referencing a previous work [27].

**Fig 6:**
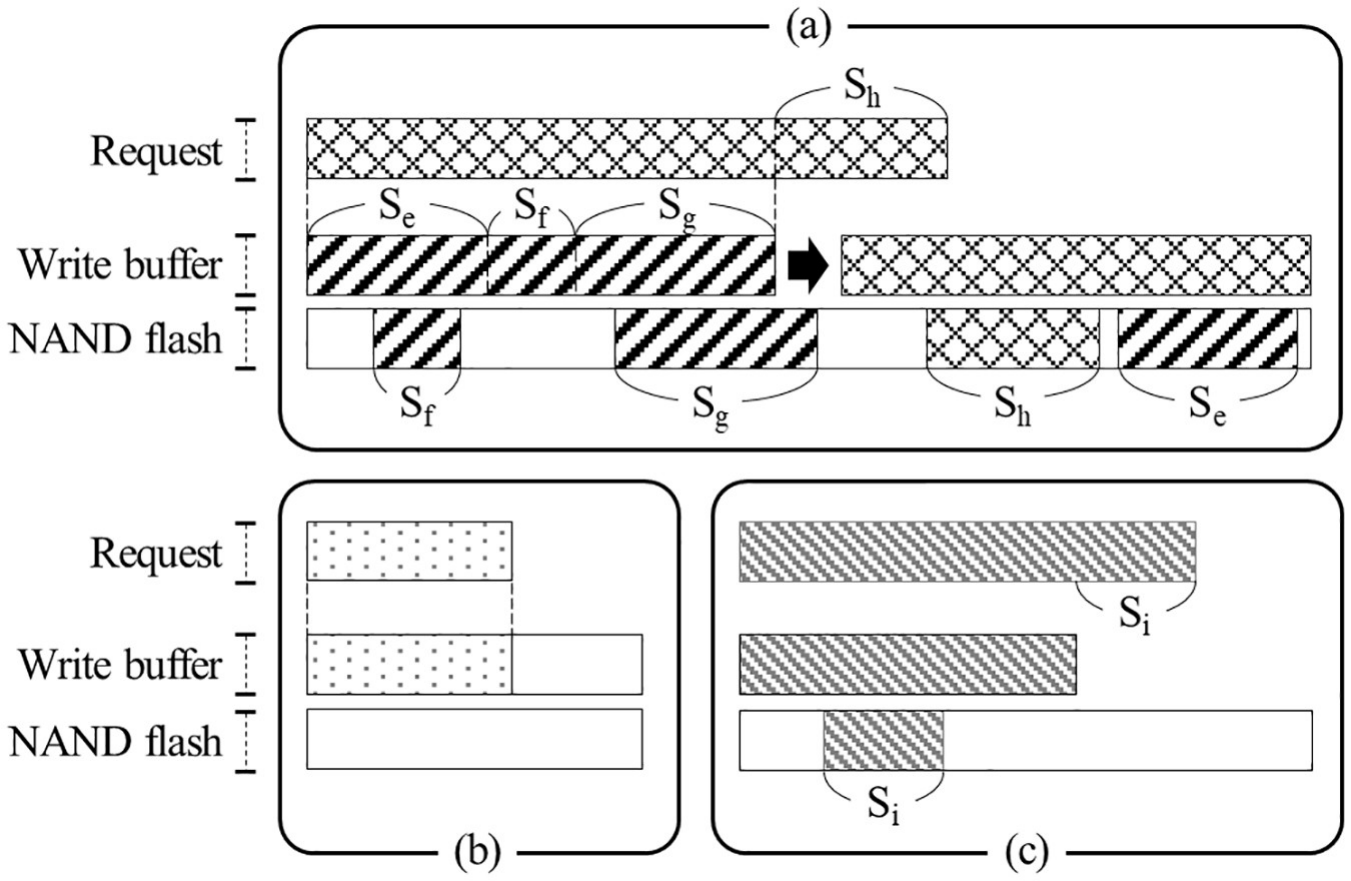
Some scenarios by write requests.

1. Open a raw DRAM-less SSD device with the O_DIRECT flag to bypass various caches and buffers in the operating system, as in the previous experiment.
2. Write an amount of data that is larger than the maximum write buffer size *S*_*BUFFER*_, which is large enough to fill a write buffer if one exists. If the SSD has a write buffer, the write buffer will be filled with the useless data.
3. Measure the latency incurred while writing data of specific size *s*. If there is a write buffer in the SSD, the write buffer will be already fully filled with the data written in step 2). If a write operation is requested in this situation, the write buffer will not have enough space to accommodate it, and thus at least some data should be written to NAND flash memory. Although it depends on the write buffer management algorithm in the controller, the newly written data are usually stored in the write buffer and the existing data in the write buffer are moved to NAND flash memory. If the request size exceeds the write buffer size, the overflow amount of data will be written into NAND flash memory directly (Fig 6(a)). In any case, the time taken to write the requested amount of data in NAND flash memory is measured in this step, and this time will still be the same even without the write buffer.
4. Move data in the write buffer into NAND flash memory using the *flush* command. Obviously, if there is no write buffer in the controller, this command will not do anything.
5. Repeat step 3). If the SSD has a write buffer, the latency measured in this step will be less than that of step 3) because all or part of the requested data is stored in the write buffer (Fig 6(b) and 6(c)). If SSD does not have a write buffer, the results of step 3) and this step will be similar because the same amount of data as requested for write should be eventually be written to NAND flash memory in both cases.
6. Repeat steps 2) to 5) while increasing s from *S*_*MIN*_ to *S*_*MAX*_ in steps of *S*_*INC*_. If SSD has a write buffer, the latency of the write operation will dramatically increase when the size of the data requested exceeds that of the write buffer.

**Fig 7:**
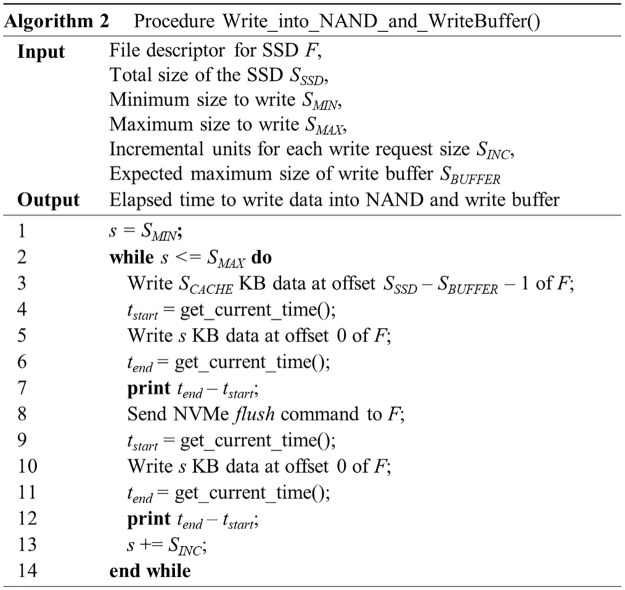
An algorithm for testing the existence of a write buffer.

The pseudocode for this test is given in Fig 7. We performed the above experiment with SSD-A, SSD-B, and SSD-C when *S*_*MIN*_, *S*_*MAX*_, and *S*_*INC*_ were 64 KB, 2,048 KB, and 2 KB, respectively. Considering the maximum configurable HMB size of SSDs used, we set *S*_*BUFFER*_ to 512 MB.

**Fig 8:**
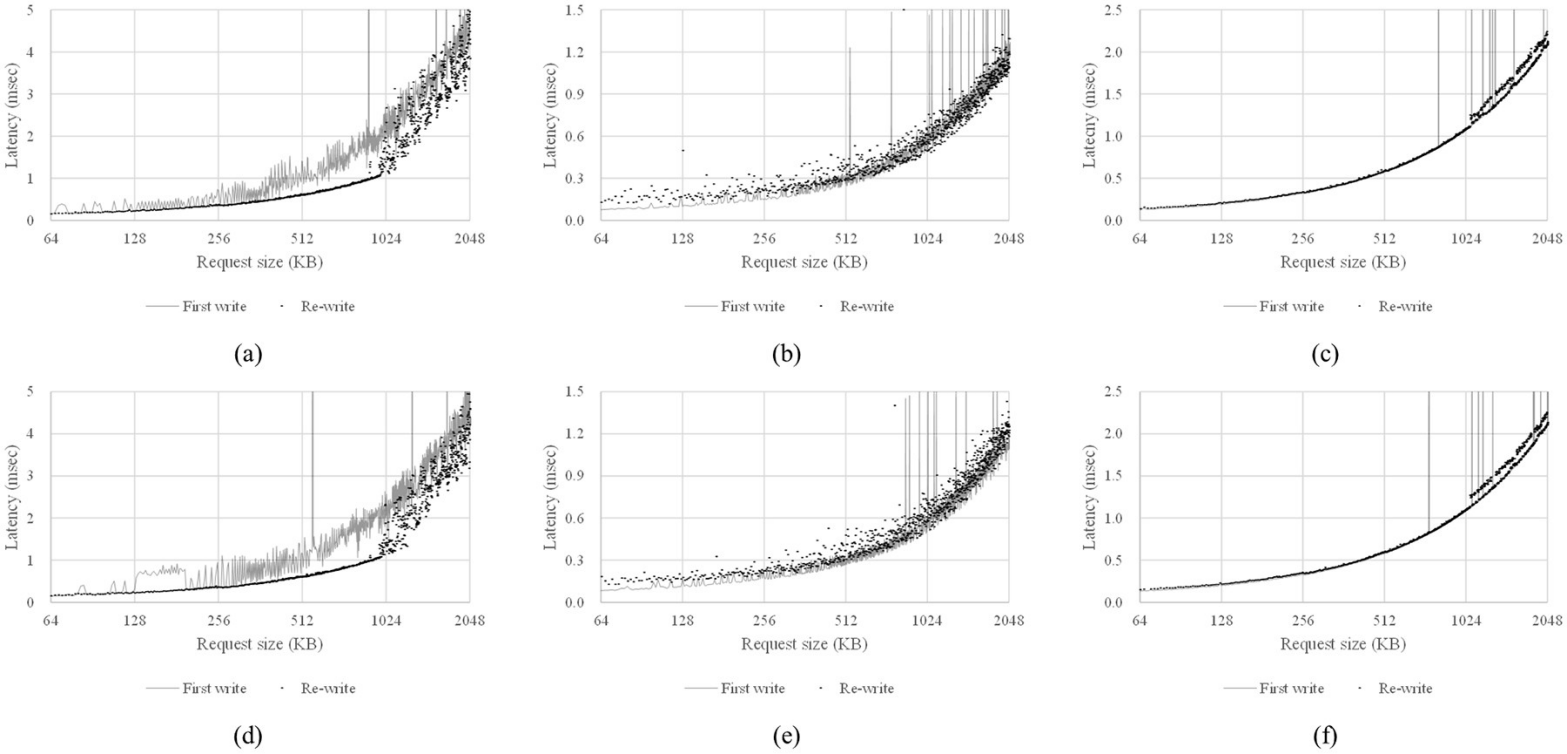
Test for existence of write buffer in the host memory buffer. (a) SSD-A, with HMB, (b) SSD-B, with HMB, (c) SSD-C, with HMB, (d) SSD-A, without HMB, (e) SSD-B, without HMB, and (f) SSD-C, without HMB.

As Fig 8(a) shows, when the request size is less than 964 KB, because all data to be written can be stored into a write buffer, the latency of the re-write operation measured in step 5) has a relatively clean curve with respect to the request size. However, if the request size is larger than 964 KB, the distribution of the latency spreads while the values also increase sharply. This is because the remaining data could not be stored in the write buffer must be written to NAND flash memory. Again, we can conclude that SSD-A uses other media instead of the HMB as a write buffer because this behavior is almost same, even when the HMB is not activated (Fig 8(d)).

In the results of SSD-B and SSD-C, there is no similarly clear behavior (Fig 8(b) and 8(c)). However, we can infer that neither SSD uses the HMB as a write buffer because there is little difference when HMB is not activated (Fig 8(e) and 8(f)). The hardware specification for SSD-C notes that it employs an SLC cache to enhance write operations [33].

### Use of HMB as a mapping table cache

Recently, many SSDs have begun to use a page-level address mapping technique, which requires 0.1% of an SSD’s total storage capacity for storing the mapping table [34]. Because the DRAM of the controller is not large enough to hold all of the mapping table entries, in general, just a part of mapping table is cached and the entire mapping table is maintained in NAND flash memory [23].

To determine whether DRAM-less SSDs use the HMB as the mapping table cache, we perform the following experiment.

1. As in the other experiments, open the raw DRAM-less SSD device with the O_DIRECT flag.
2. Erase all data in the SSD and then write data sequentially up to the capacity of the SSD to create a new mapping table. Wait long enough for the SSD to complete the internal operations generated by erase and write. Because some SSDs cannot properly process the *format* command, which erases data in the SSDs, we used the *dataset management* command with the deallocate option, which is like the *trim* command of SATA for erasing data in an SSD.
3. Divide the entire space of SSD into *d* sections and read 512 B from the beginning position of each section, that is, 0, *S*_*ssd*_*/d*, 2*(S_*ssd*_*/d)*, 3*(S_*ssd*_*/d)*, … (d-1)*(S_*ssd*_*/d)* while measuring the elapsed time. Because we only focus on operations for managing the address mapping table in this experiment, we set the request size to 512 B, which is the minimum read request size, to reduce the effects of a data read. Because we have sequentially written data into the entire SSD in step 2), the mapping table entries for all data in the SSD would have been completely created. If the HMB is used to cache the mapping table entries and has enough free space for caching them, the mapping table entries for the beginning position of each section should be cached in this step.
4. Repeat step 3) while increasing *d* from *D*_*MIN*_ to *D*_*MAX*_ in steps of *D*_*INC*_ and measure the time taken for processing the read request in each section. As *d* increases, the number of mapping table entries to be cached also increases. If *d* exceeds the limit of the mapping table entries that can be cached in the HMB, the time for processing each read request will dramatically increase because of cache misses.

**Fig 9:**
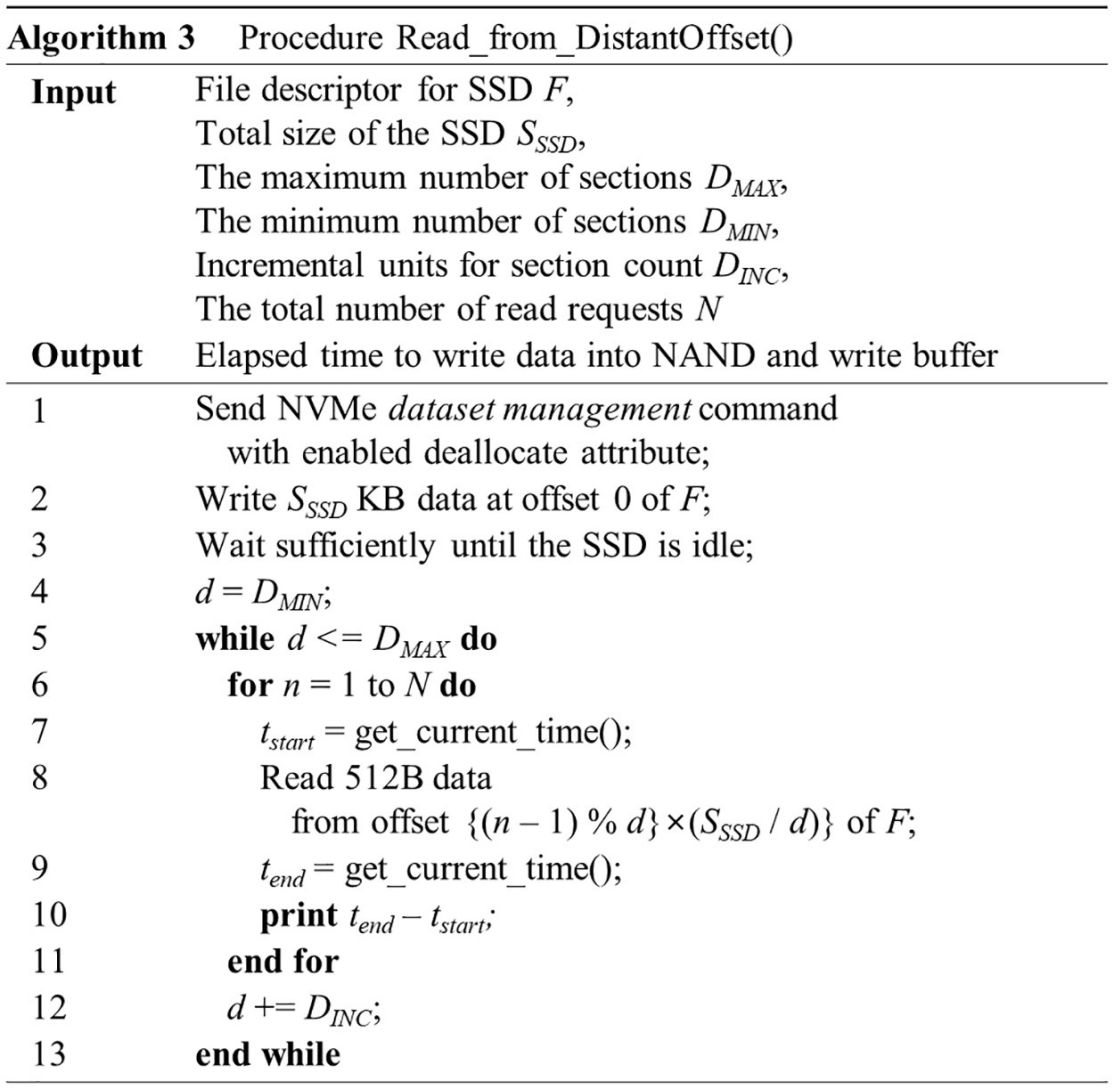
An algorithm for testing the existence of a mapping table cache.

The pseudocode for this test is given in Fig 9. For this experiment, we first set *D*_*MIN*_, *D*_*MAX*_, and *D*_*INC*_ to 100, 20,000, and 100, respectively. While SSD-A and SSD-C can allocate from 8 MB to 480 MB for the HMB, SSD-B can allocate only 64 MB.

**Fig 10:**
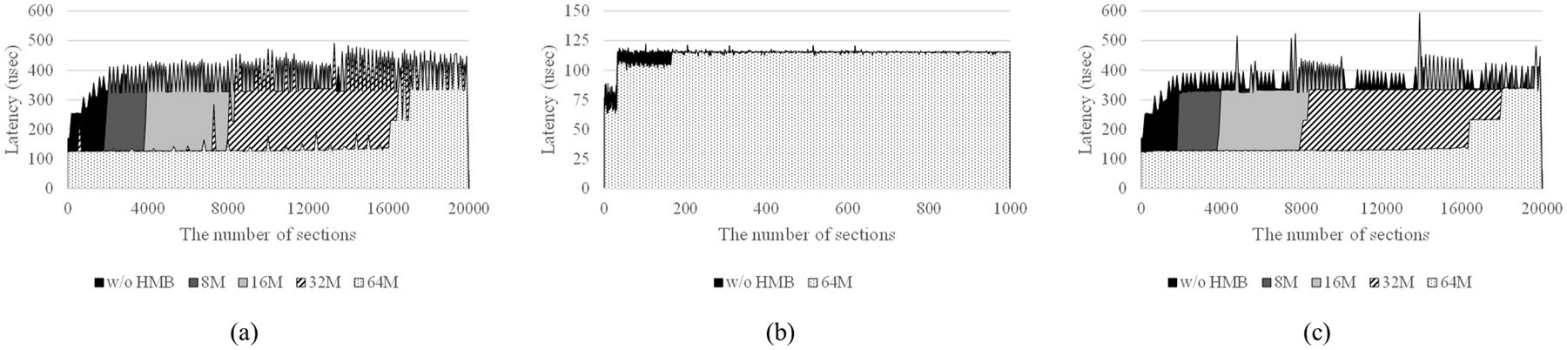
Test for existence of mapping table cache in HMB. (a) SSD-A, (b) SSD-B, and (c) SSD-C.

For SSD-A and SSD-C, the latency sharply increases when *d* is 2,000, 4,000, 8,000, and 16,000 with respect to HMB size (Fig 10(a) and 10(c)). When HMB is not used, the SSDs show high latency in general. As a result, we can guess that SSD-A and SSD-C store the address mapping table entries in the HMB. Because the HMB size and number of sections when the latency sharply increases are proportional, we can conclude that part of the HMB is used as a mapping table cache. If both SSDs use the HMB only for the mapping table cache, the size of caching unit for storing mapping table in the HMB can be estimated to be about 4 KB. For SSD-B, when the number of sections is less than 200, we can see a small difference in the latency when using and not using the HMB. However, we cannot conclude that SSD-B uses the HMB as mapping table cache from this observation alone because it is not possible to experiment with different HMB sizes.

## Conclusion and future work

In this paper, we studied the effects and roles of the HMB in commercial DRAM-less SSDs that support the HMB feature. We first experimentally showed that commercial DRAM-less SSDs have worse I/O performance than SSDs with internal DRAM, but they can improve it when using the HMB feature of NVMe. Because the internals of SSDs is usually not known, we also presented several methods that can analyze experimentally how the HMB is used to improve the I/O performance in DRAM-less SSDs. Experimental results show that DRAM-less SSDs used in our works mainly use the HMB for caching the address mapping table rather than the read cache or write buffer.

These results will be useful for the further development and utilization of DRAM-less SSDs by the wider research community. We believe that the HMB feature can provide many opportunities for performance improvement in DRAM-less SSDs. Especially, as the HMB is shared by both the host and the SSD device, the I/O performance can be significantly improved if they cooperate to use the HMB efficiently. We are now studying how to optimize the I/O software stack from file systems to FTL within SSDs by using the HMB for I/O performance improvement.
